# The complete mitochondrial genome of *Hermetia illucens* (Diptera: Stratiomyidae)

**DOI:** 10.1080/23802359.2017.1307708

**Published:** 2017-03-29

**Authors:** Yingju Qi, Jingyang Xu, Xiaoxuan Tian, Yichuan Bai, Xishu Gu

**Affiliations:** a Tianjin State Key Laboratory of Modern Chinese Medicine, Tianjin University of Traditional Chinese Medicine, Tianjin, China;; b Research and Development Center of TCM, Tianjin International Joint Academy of Biotechnology & Medicine, Tianjin, China;; c Tianjin Institute of Plant Protection, Tianjin, China

**Keywords:** *Hermetia illucens*, mitochondrial genome, Stratiomyidae

## Abstract

*Hermetia illucens*, one kind of necrophagous insects, belongs to the family Stratiomyidae (Diptera). In this study, the complete mitochondrial genome of *H. illucens* was investigated. The total number of nucleic acids were 15,698 bp. The genome contains 13 protein-coding genes, 22 tRNA genes, 2 rRNA genes, and a non-coding control region. The phylogenetic relationship of *H. illucens* and its 12 related species was reconstructed to confirm the taxonomic status of our sample.

*Hermetia illucens* belongs to the family Stratiomyidae. It is one sort of necrophagous insects originating from America (Yang et al. 2016). Their larvae have many medicinal values such as anti-inflammatory, analgesia, and haematolysis (Li 2016). Complete mitochondrial genome has been considered as a useful tool for population genetic and phylogenetic studies (Cameron 2014). The complete mitochondrial genome of *H. illucens* was first reported in this paper.

In this study, the individuals of *H. illucens* were provided by the Tianjin Institute of Plant Protection located in Tianjin city, China (voucher number: hsm-1). We used the high-throughput sequencing method to acquire the *H. illucens* complete mitochondrial genome sequences (GenBank accession number: KY679159). The complete genome is 15,698bp in length. The composition of the whole genome is 35.1% A, 39.3% T, 19.6% C, and 6.0% G. It contains 13 protein-coding genes (PCGs), 22 tRNA genes, and 2 rRNA genes. The longest gene within this molecule is *ND5* containing 1719bp and the shortest is *ATP8* gene which is 168 bp. Seven PCGs start from ATG, and five PCGs start from ATT while *COX1* starts from TCG. *ND5* and *CytB* use TAG as stop codon, and at the same time, *ND1* uses incomplete stop codon, i.e. T––. Besides that, the other PCGs use TAA as stop codon. tRNAs were predicted by MITOS Web Server based on their cloverleaf secondary structure. As shown in Figure 1, the phylogenetic analysis among *H. illucens* and its related species was conducted.

**Figure 1.**
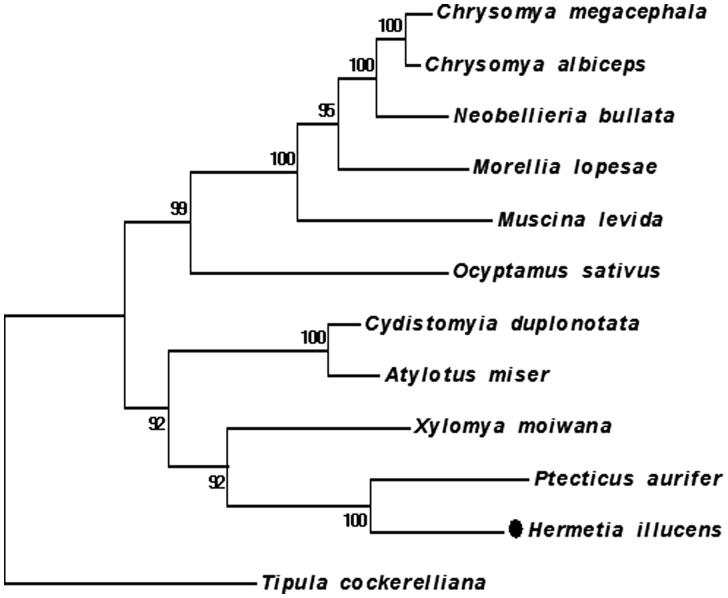
Twelve mitochondrial DNA were obtained from GenBank to build the maximum-likelihood phylogenetic tree using MEGA (version 5.10) based on sequences of translated mitochondrial proteins. MUSCLE was used to align the sequences. 1000 replicates of bootstrap were set. Sequence data used in the study are the following: *Ptecticus aurifer*, KT225297.1; *Xylomya moiwana*, KT225302.1; *Muscina levida*, KT272866.1; *Morellia lopesae*, KT272863.1; *Chrysomya megacephala*, KT272865.1; *C. albiceps*, KT272864.1; *Ocyptamus sativus*, KT272862.1; *Neobellieria bullata*, KT272859.1; *Atylotus miser*, NC_030000.1; *Cydistomyia duplonotata*, NC_008756.1; *Tipula cockerelliana*, NC_030520.1.
